# Plasma Neurofilament Light Chain (NF-L) Is a Prognostic Biomarker for Cortical Damage Evolution but Not for Cognitive Impairment or Epileptogenesis Following Experimental TBI

**DOI:** 10.3390/ijms232315208

**Published:** 2022-12-02

**Authors:** Mette Heiskanen, Olli Jääskeläinen, Eppu Manninen, Shalini Das Gupta, Pedro Andrade, Robert Ciszek, Olli Gröhn, Sanna-Kaisa Herukka, Noora Puhakka, Asla Pitkänen

**Affiliations:** 1 A.I. Virtanen Institute for Molecular Sciences, University of Eastern Finland, P.O. Box 1627, 70211 Kuopio, Finland; 2 Institute of Clinical Medicine/Neurology, University of Eastern Finland, P.O. Box 1627, 70211 Kuopio, Finland; 3 Department of Neurology, Kuopio University Hospital, P.O. Box 1777, 70211 Kuopio, Finland

**Keywords:** fluid-percussion injury, post-traumatic epilepsy, rat, ROC analysis, single molecule array (SIMOA)

## Abstract

Plasma neurofilament light chain (NF-L) levels were assessed as a diagnostic biomarker for traumatic brain injury (TBI) and as a prognostic biomarker for somatomotor recovery, cognitive decline, and epileptogenesis. Rats with severe TBI induced by lateral fluid-percussion injury (n = 26, 13 with and 13 without epilepsy) or sham-operation (n = 8) were studied. During a 6-month follow-up, rats underwent magnetic resonance imaging (MRI) (day (D) 2, D7, and D21), composite neuroscore (D2, D6, and D14), Morris-water maze (D35–D39), and a 1-month-long video-electroencephalogram to detect unprovoked seizures during the 6th month. Plasma NF-L levels were assessed using a single-molecule assay at baseline (i.e., naïve animals) and on D2, D9, and D178 after TBI or a sham operation. Plasma NF-L levels were 483-fold higher on D2 (5072.0 ± 2007.0 pg/mL), 89-fold higher on D9 (930.3 ± 306.4 pg/mL), and 3-fold higher on D176 32.2 ± 8.9 pg/mL after TBI compared with baseline (10.5 ± 2.6 pg/mL; all *p* < 0.001). Plasma NF-L levels distinguished TBI rats from naïve animals at all time-points examined (area under the curve [AUC] 1.0, *p* < 0.001), and from sham-operated controls on D2 (AUC 1.0, *p* < 0.001). Plasma NF-L increases on D2 were associated with somatomotor impairment severity (ρ = −0.480, *p* < 0.05) and the cortical lesion extent in MRI (ρ = 0.401, *p* < 0.05). Plasma NF-L increases on D2 or D9 were associated with the cortical lesion extent in histologic sections at 6 months post-injury (ρ = 0.437 for D2; ρ = 0.393 for D9, *p* < 0.05). Plasma NF-L levels, however, did not predict somatomotor recovery, cognitive decline, or epileptogenesis (*p* > 0.05). Plasma NF-L levels represent a promising noninvasive translational diagnostic biomarker for acute TBI and a prognostic biomarker for post-injury somatomotor impairment and long-term structural brain damage.

## 1. Introduction

Every year, approximately 2.5 million people suffer traumatic brain injury (TBI) in Europe (https://www.center-tbi.eu/, accessed on 4 October 2022) and the United States (https://www.cdc.gov/traumaticbraininjury/, accessed on 4 October 2022), with over 60 million affected globally [1]. Despite the demonstrated efficacy of a large number of interventions in preclinical proof-of-concept trials for mitigating the secondary damage and consequent functional deficits of TBI, including cognitive decline and epileptogenesis, none of these interventions have advanced to clinical application [2,3,4]. The development of treatments for TBI and its consequent morbidities remains a major unmet medical need [5]. One major obstacle to efficient therapy development is the lack of preclinical and clinical biomarkers that could be used to stratify subjects for therapy trials and monitor treatment effects [5,6].

Blood-derived biomarkers are proposed as minimally invasive tools for the stratification of study subjects as well as for monitoring therapy responses, for example, in Alzheimer’s disease [7]. In patients with TBI, biofluid biomarkers, including glial fibrillary acidic protein (GFAP), ubiquitin C-terminal hydrolase-L1 (UCH-L1), s100β, and neurofilaments, show some promise for diagnosing injury severity, monitoring disease progression, and predicting the structural and functional outcome and therapy response [3,8,9]. Compared with humans, blood and cerebrospinal fluid (CSF) biomarker studies in animal models of TBI remain sparse [8]. This is a major knowledge gap, as the use of biomarkers could speed up laborious in vivo experimental studies, allowing for more rigorous, controlled, and cost-effective therapy discovery [5,10].

Neurofilament light chain (NF-L) is a neuron-specific cytoskeletal protein that provides structural support to axons and dendrites, but it is also found in synapses, where it is thought to influence the distribution of NMDA GluN1 receptors [11,12]. NF-L is one of the five subunits that form the full neurofilament, the other four being neurofilament heavy and medium chains, α-internexin, and peripherin [12]. Neurofilaments are exclusively expressed in neurons, which makes them specific indicators of neuroaxonal damage [11]. Accordingly, acute and chronic neuro-axonal damage due to TBI or other brain diseases triggers the release of a large quantity of NF-L from neurons to the interstitial fluid, from which it enters the cerebrospinal fluid (CSF) and blood [12,13,14]. Importantly, recent studies have demonstrated that NF-L levels in the blood and CSF at acute post-injury time-points are associated with TBI severity and predict clinical outcome, including progression of structural brain damage, functional recovery, and death [15,16,17,18,19].

Animal models can recapitulate various long-term clinically relevant structural and functional abnormalities of TBI, including cognitive impairment and post-traumatic epilepsy (PTE) [20,21]. In the present study, we aimed (a) to determine the temporal profile of circulating NF-L, (b) to assess associations between plasma NF-L levels and the evolution of cortical damage, and (c) to determine whether NF-L could be used as a sensitive and specific prognostic biomarker to predict the functional outcome after TBI, focusing on somatomotor recovery, cognitive decline, and epileptogenesis.

## 2. Results

### 2.1. Sample Quality and Lack of a Hemolysis Effect on Plasma NF-L Levels

Absorbance at 414 nm measured from plasma samples with NanoDrop varied from 0.08 to 0.65 (*n* = 120, mean 0.23 ± 0.10, median 0.21). Of the 120 samples, 39 (33%) had an absorbance ≥ 0.25 and were considered to be hemolyzed. No correlation, however, was detected between absorbance at 414 nm and plasma NF-L levels at any time-point (Spearman correlation, *p* > 0.05 for all). Consequently, no samples were excluded due to hemolysis.

### 2.2. Post-TBI Increase in Plasma NF-L Levels Is Time-Dependent

**Figure 1:**
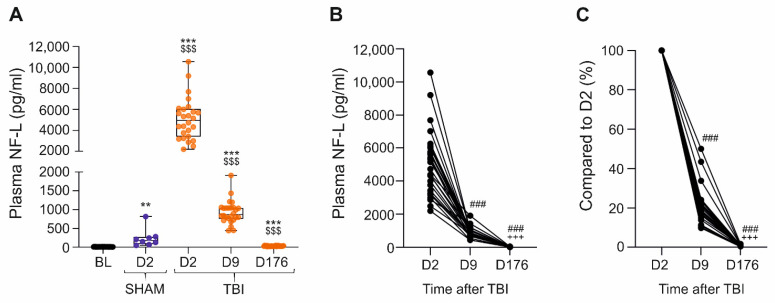
Plasma NF-L concentrations at different time-points after a sham operation or TBI. (**A**) Box and whisker plots (whiskers: minimum and maximum; box: interquartile range; line: median) showing plasma NF-L levels (*y*-axis) in different groups (*x*-axis). Samples collected at baseline (n = 34) before any operation (BL on D-6) were considered comparable to naïve samples. In the sham group (n = 8, blue), plasma NF-L levels were analyzed on D2 post-operation only. In the TBI group (n = 26, orange), plasma NF-L levels were assessed on D2, D9, and D176 after TBI. Each dot represents 1 animal. Note the slightly elevated plasma NF-L levels in the sham-operated animals on D2 compared with their baseline values (** *p* < 0.01, Wilcoxon). In the TBI group, the NF-L levels were elevated on all testing days as compared to their baseline values [Friedman test (*p* < 0.001) followed by *post hoc* analysis with Wilcoxon: ***, *p* < 0.001]. On D2 and D9, the average NF-L levels were higher in the TBI group than in the sham group ($$$, *p* < 0.001, Mann-Whitney U test). (**B**) Dynamics of NF-L concentrations in individual TBI animals over time [Friedman test (*p* < 0.001) followed by *post hoc* analysis with Wilcoxon: ###, *p* < 0.001 compared with D2; +++, *p* < 0.001 compared with D9]. (**C**) Change in plasma NF-L levels as a percentage over time (D2 marked as 100%) [Friedman test (*p* < 0.001) followed by *post hoc* analysis with Wilcoxon: ###, *p* < 0.001 compared with D2; +++, *p* < 0.001 compared with D9]. Abbreviations: BL, baseline; D2, day 2 after TBI; D9, day 9; D176, day 176 (6 months); NF-L, neurofilament light chain; TBI, traumatic brain injury.

***Baseline (Naïve).*** Plasma NF-L levels at different post-TBI time points are summarized in Figure 1A. At baseline, the plasma NF-L levels varied from 4.2 pg/mL to 16.0 pg/mL (*n* = 34; mean 10.3 + 2.9 pg/mL; median 10.3 pg/mL).

***Sham.*** On D2 (48 h after sham-operation), the plasma NF-L levels were increased in craniotomized sham-operated experimental controls compared with the baseline levels of the same animals (244.3 ± 245.6 pg/mL vs. 9.6 ± 2.9 pg/mL, *p* < 0.01) (Figure 1A).

***TBI.*** On D2, injured animals showed a 483-fold increase in the mean plasma NF-L levels compared with the baseline levels of the same rats (5072.0 ± 2007.0 pg/mL vs. 10.5 ± 2.6 pg/mL, *p* < 0.001) and a 21-fold increase compared with the sham-operated group (5072.0 ± 2007.0 pg/mL vs. 244.3 ± 245.6 pg/mL, *p* < 0.001) (Figure 1A).

On D9, the plasma NF-L levels in the TBI group decreased to 930.3 ± 306.4 pg/mL. The levels remained elevated when compared to the same rats’ baseline levels (930.3 ± 306.4 pg/mL vs. 10.5 ± 2.6 pg/mL, *p* < 0.001) or to the sham-operated controls on D2 (930.3 ± 306.4 pg/mL vs. 244.3 ± 245.6 pg/mL, *p* < 0.001) (Figure 1A).

On D176 (6 months post-TBI), NF-L levels remained approximately 3-fold higher than the baseline levels of the same animals (32.2 ± 8.9 pg/mL vs. 10.5 ± 2.6 pg/mL, *p* < 0.001), but only 13% of that in the sham group on D2 (32.2 ± 8.9 pg/mL vs. 244.3 ± 245.6 pg/mL, *p* < 0.001) (Figure 1A).

The temporal dynamics of the plasma NF-L concentration in each TBI rat are summarized in Figure 1B. The percent change in plasma NF-L levels from D2 to D9 is summarized in Figure 1C. On average, the plasma NF-L concentration on D9 was 20.4 ± 9.5% (range 9.80–50.1%) of that on D2. That is, the plasma NF-L concentration decreased by approximately 80% from D2 to D9. On D176, plasma NF-L levels were only 0.7 ± 0.4% (range 0.3–1.7%) of the levels on D2.

### 2.3. Plasma NF-L Levels Correlated with the Severity of Acute and Chronic Cortical Damage

To assess whether plasma NF-L levels correlated with the severity of the lateral fluid-percussion injury (FPI)-induced cortical lesion at acute and chronic time-points, we measured (a) the volume of abnormal cortical T_2_ in MRI on D2, D7, and D21 post-injury and (b) the cortical lesion area in unfolded cortical maps on D182 post-injury.

#### 2.3.1. Plasma NF-L and Volume of Abnormal Cortical T_2_ in MRI

Quantitative T_2_ MRI was available for all rats included in the NF-L analysis.

**Figure 2:**
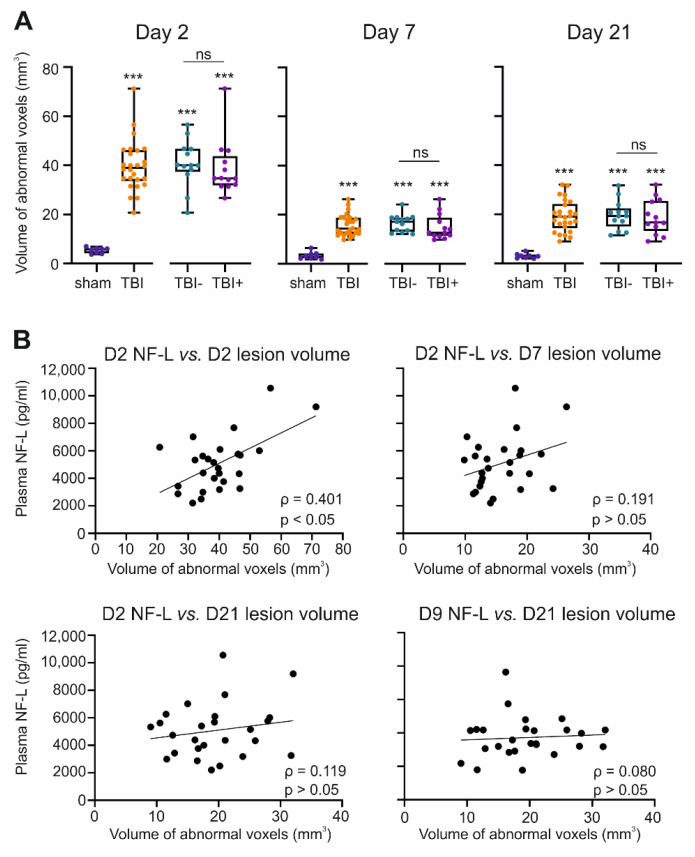
Plasma NF-L and cortical lesion severity in quantitative T_2_ magnetic resonance imaging (MRI). (**A**) Box and whisker plots (whiskers: minimum and maximum; box: interquartile range; line: median) showing the total volume of abnormal pixels (cortical T_2_ signal, *y*-axis) in rat brain MRI on D2, D7, and D21 after TBI (n = 26) or sham operation (n = 8). The TBI group included 13 rats without epilepsy (TBI−) and 13 rats with epilepsy (TBI+). Each dot represents 1 animal. (**B**) Spearman correlation between the plasma levels of NF-L (*y*-axis) and volume of abnormal T_2_ area (*x*-axis) in MRI (TBI group only) on D2, D7, and D21. Note that on D2, the higher the NF-L level, the greater the volume of the abnormal T_2_ area. No correlations were detected at later time-points. Statistical significance: ***, *p* < 0.001 as compared with the sham group (Mann-Whitney U test). Abbreviations: D2, day 2 after TBI; D9, day 9; D21, day 21; NF-L, neurofilament light chain; ns, not significant; TBI, traumatic brain injury.

***Sham.*** On D2, the cortical volume of the abnormal T_2_ area was small (5.4 ± 1.2 mm^3^, range 3.8 mm^3^–7.0 mm^3^, median 5.7 mm^3^). On D7, the volume of the T_2_ change was 3.3 ± 1.5 mm^3^ and on D21, 3.1 ± 1.0 mm^3^ (Figure 2A). The ipsilateral signal increase on D2, D7, and D21 was located in areas close to the rhinal fissure at the rostrocaudal level—3.5 mm from the bregma—as the median of the ipsilateral T_2_ area was higher than that contralaterally. A parasagittal signal decrease was observed on D7.

On D2, plasma NF-L levels did not correlate with the volume of abnormal T_2_ in the sham group (*n* = 8, ρ = 0.095, *p* > 0.05).

***TBI.*** On D2, the mean volume of the abnormal cortical T_2_ area was 39.8 ± 10.4 mm^3^ (Figure 2A). By D7, the volume of the abnormal T_2_ area had decreased to 15.8 ± 4.4 mm^3^ (*p* < 0.001 compared with D2). On D21, the volume of the abnormal cortical T_2_ area was 19.4 ± 6.4 mm^3^ (*p* < 0.001 compared with D2 or D7).

On D2, the higher the plasma NF-L concentration, the greater the volume of the abnormal cortical T_2_ area (ρ = 0.401, *p* < 0.05) (Figure 2B). No correlation was detected between the D2 plasma NF-L levels and lesion volumes on D7 or D21 (*p* > 0.05). Also, no correlation was detected between the plasma NF-L levels on D9 and the volume of the abnormal T_2_ area on D7 or D21 (D7 ρ = 0.127, *p* > 0.05; D21 ρ = 0.080, *p* > 0.05) (Figure 2B).

#### 2.3.2. Plasma NF-L and Cortical Lesion Area in Histologic Sections

None of the SIMOA cohort rats had abscesses or other non-TBI-related lesions. The cortical lesion area in unfolded maps prepared from histologic section D182 post-TBI ranged between 14.0–56.9 mm^2^ (median 33.4 mm^2^) (Supplementary Figure S1). There was no difference in the cortical lesion area between the TBI+ (31.5 ± 13.3 mm^2^) and TBI− (37.7 ± 10.6 mm^2^) groups (*p* > 0.05).

A correlation analysis revealed that the higher the plasma NF-L level on D2 or D9, the larger the cortical lesion area on D182 (ρ = 0.437 for D2 and ρ = 0.393 for D9, *p* < 0.05 for both). In contrast, no correlation was detected between plasma NF-L levels on D176 and the cortical lesion area on D182 (*p* > 0.05).

### 2.4. Plasma NF-L as a Diagnostic Biomarker for Sham-Operation and TBI

Next, we assessed whether plasma NF-L levels on D2, D9, or D176 after TBI differentiated rats with TBI from naïve animals and/or sham-operated controls.

**Figure 3:**
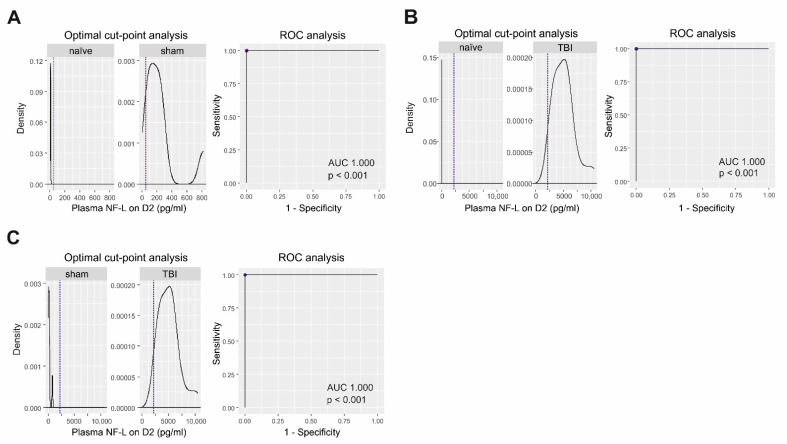
Plasma NF-L as a diagnostic biomarker for sham operation and TBI. (**A**) ROC analysis indicated that NF-L levels on D2 distinguished sham-operated rats (n = 8) from the naïve condition (baseline samples, n = 34) with 100% sensitivity and 100% specificity (AUC 1.0, *p* < 0.001, cut-off 49 pg/mL (dashed line)]. (**B**) NF-L levels on D2 distinguished TBI rats (n = 26) from the naïve condition (n = 34) with 100% sensitivity and 100% specificity (AUC 1.0, *p* < 0.001, cut-off: 2201 pg/mL). (**C**) NF-L levels on D2 distinguished TBI (n = 13) and sham-operated rats (n = 13) with 100% sensitivity and 100% specificity (AUC 1.0, *p* < 0.001, cut-off: 2201 pg/mL). Abbreviations: AUC, area under the curve; BL, baseline; D2, day 2 post-TBI, NF-L, neurofilament light chain; ROC, receiver operating characteristics; TBI, traumatic brain injury.

***Sham-operated experimental controls vs. naïve (baseline) samples.*** On D2, ROC analysis revealed that plasma NF-L levels differentiated sham-operated experimental controls from naïve animals with 100% sensitivity and 100% specificity (AUC 1.0, *p* < 0.001; cut-off 49.1 pg/mL) (Figure 3A).

***TBI vs. naïve (baseline) samples.*** On D2, NF-L levels differentiated TBI animals from naïve rats with 100% sensitivity and 100% specificity (AUC = 1.0, *p* < 0.001; cut-off: 2201 pg/mL) (Figure 3B). On D9, plasma NF-L levels differentiated TBI animals from naïve rats with 100% sensitivity and 100% specificity (AUC = 1.0, *p* < 0.001; cut-off 442 pg/mL). Even on D176, plasma NF-L levels differentiated TBI animals from naïve rats with 100% sensitivity and 97% specificity (AUC = 0.999, *p* < 0.001; cut-off 15.8 pg/mL).

***TBI vs. sham-operated controls.*** On D2, plasma NF-L levels differentiated the TBI and sham-operated animals with 100% sensitivity and 100% specificity (AUC 1.0, *p* < 0.001; cut-off 2201 pg/mL) (Figure 3C).

### 2.5. Plasma NF-L as a Prognostic Biomarker for Somatomotor Recovery

***Plasma NF-L and neuroscore.*** In sham-operated experimental controls (n = 8), the composite neuroscore differed between testing days over the 14-day follow-up (average neuroscore: D2 26.5, D6 27.4, D14 27.5, Friedman test, *p* < 0.01). *Post hoc* analysis with the Wilcoxon test revealed improvement in the neuroscore from D2 to D6 (*p* < 0.05), but no difference between D6 and D14 (*p* > 0.05).

**Figure 4:**
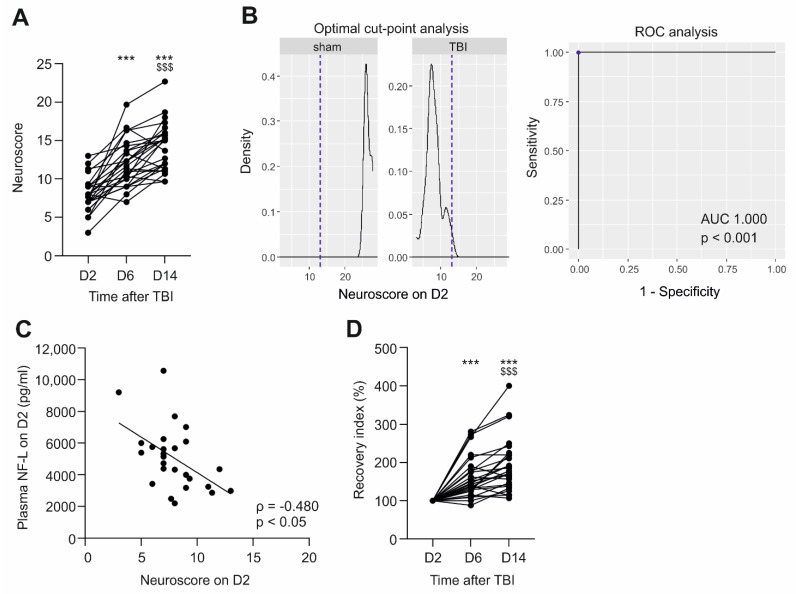
Plasma NF-L and somatomotor performance after TBI. (**A**) Evolution of the neuroscore in each TBI rat (n = 26) over the 14-day follow-up (D2, D6, and D14) after TBI. Note that in the sham-group, the average neuroscore was 26.5 on D2, 27.4 on D6, and 27.5 on D14. (**B**) The optimal cut-off and ROC analysis indicated that the D2 neuroscore differentiated TBI (n = 26) and sham-operated rats (n = 8) with 100% sensitivity and 100% specificity [AUC 1.0, *p* < 0.001, cut-off 13.0 (dashed line)]. (**C**) Spearman’s correlation revealed that the higher the plasma NF-L levels on D2, the lower the neuroscore on D2 post-TBI (ρ = −0.480, *p* < 0.05). (**D**) Improvement of the neuroscore (recovery index) for each TBI rat (n = 26), compared with the neuroscore on D2. Statistical significances: ***, *p* < 0.001 compared to D2; $$$, *p* < 0.001 compared to D6 (Wilcoxon matched pairs signed rank test). Abbreviations: AUC, area under the curve; D2, day 2 post-TBI; D6, day 6, D14, day 14; NF-L, neurofilament light chain; ROC, receiver operating characteristics; TBI, traumatic brain injury.

In the TBI group, the evolution of the composite neuroscore over the 14-day testing period is shown in Figure 4A (Friedman test, *p* < 0.001, followed by Wilcoxon test). On D2, the average neuroscore was 7.9 (range 3.0–13.0, median 7.8), on D6 12.0 (range 7.0–19.7, median 11.5; *p* < 0.001 compared with D2), and on D14 14.2 (range 9.7–22.7, median 15.0; *p* < 0.001 compared with D2 and D6).

On D2, the higher the plasma NF-L concentration, the lower the neuroscore (ρ = −0.480, *p* < 0.05) (Figure 4C). Interestingly, plasma NF-L levels on D2 did not correlate with the neuroscore at later time-points (*p* > 0.05).

***Plasma NF-L and recovery index.*** To assess whether plasma NF-L levels differed between rats with poor or good recovery, we next calculated the recovery index for each animal. The mean D14/D2 neuroscore recovery index in the TBI group was 193% ± 71% (range 107–400%). Of the 26 rats with TBI, 9 had a D14/D2 recovery index greater than 200% (good long-term recovery), and 17 had an index ≤ 200% (poor long-term recovery). The mean D6/D2 recovery index was 161% ± 53% (range 88–281%, Figure 4D). Of the 26 rats, 12 had a D6/D2 recovery index greater than 150% (early recovery) and 14 had an index ≤ 150% (no early recovery). The mean D14/D6 recovery index was 121% ± 24% (range 82–180%). Of the 26 rats, 21 had D14/D6 recovery index greater than 100% (late recovery), and 5 had an index ≤ 100% (no late recovery).

The plasma NF-L levels did not differ significantly on D2, D9, or D176 between rats with good or poor overall recovery (*p* > 0.05 in all). Similarly, no differences in plasma NF-L levels were detected between rats with or without early recovery or between rats with or without late recovery (all *p* > 0.05).

No correlation was detected between the plasma NF-L levels on D2, D9, or D176 and any of the recovery indices (*p* > 0.05 for all).

ROC analysis indicated that the D2, D9, or D176 plasma NF-L levels did not distinguish the good from the poor overall recovery groups, the early recovery groups from the non-early recovery groups, or the late recovery groups from the non-late recovery groups (*p* > 0.05).

### 2.6. Plasma NF-L as a Prognostic Biomarker for Memory Impairment

**Figure 5:**
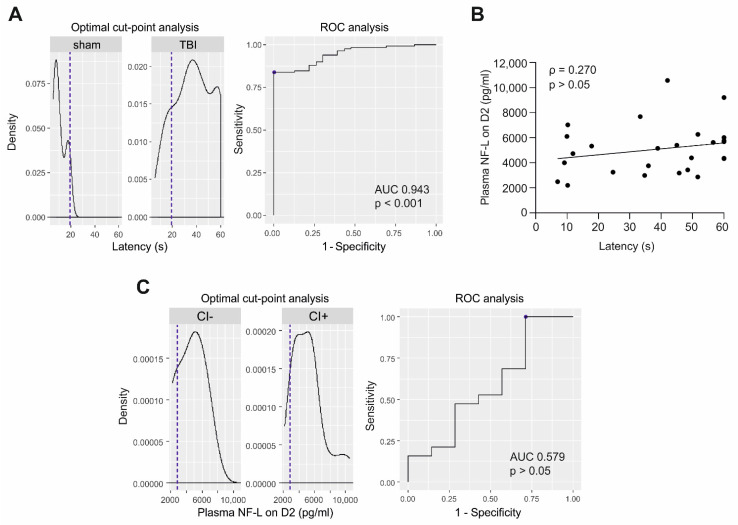
Plasma NF-L and cognitive impairment after TBI. (**A**) Optimal cut-point analysis was performed in the EPITARGET animal cohort to identify parameters that differentiate cognitively impaired (CI+) and non-impaired (CI−) rats. The cut-point latency of 19.2 s (i.e., latency to reach the platform in the Morris water maze (MWM) on the 3rd day of testing, i.e., on D37, dashed line) differentiated TBI (n = 118) and sham-operated rats (n = 23) with AUC 0.94 (*p* < 0.001) and was set as a limit for cognitive impairment. (**B**) No correlation was detected between plasma NF-L levels on D2 and MWM latency on D37 (Spearman correlation rho (ρ) = 0.270, *p* > 0.05, n = 26). (**C**) Accordingly, ROC analysis indicated that plasma NF-L levels did not distinguish CI+ (latency > 19.2 s) from CI− (<19.2 s) rats (AUC 0.58, *p* > 0.05, Mann-Whitney U test). Abbreviations: AUC, area under the curve; D2, day 2 post-TBI; ROC, receiver operating characteristics; TBI, traumatic brain injury.

***Cut-point analysis.*** Next, we assessed whether plasma NF-L levels in the early post-injury phase would predict cognitive impairment (CI). Therefore, we performed a cut-point analysis of Morris water-maze data of the entire EPITARGET animal cohort, including 23 sham-operated and 118 rats with TBI, to identify the best parameter that could be used to differentiate cognitively impaired (CI+) from non-impaired (CI−) animals [22]. The analysis revealed that a latency cut-off value of 19.2 s to reach the platform on the third testing day (D37 post-TBI) separated TBI animals from sham-operated experimental controls with an AUC of 0.94 (84% sensitivity, 100% specificity, *p* < 0.001) (Figure 5A). Latencies for rats included in the NF-L analysis are presented in Supplementary Figure S2.

In the entire EPITARGET cohort, 70% (98/141) of the rats with TBI were categorized into the “cognitively impaired” (CI+) and 30% (20/141) were categorized into the “cognitively non-impaired” (CI−) group. The percentages in the NF-L cohort were comparable, as 73% (19/26) of the TBI animals were classified into the CI+ group (latency > 19.2 s) and 27% (7/26) into the CI− group (latency < 19.2 s, performing closer to the control level) (Supplementary Figure S3A).

***Plasma NF-L levels and CI.*** Plasma NF-L levels did not differ significantly between CI− and CI+ rats at any time-point (*p* > 0.05) (Supplementary Figure S3B).

No correlation was detected between D2 plasma NF-L levels and latency on D37 in the TBI group (ρ = 0.270, *p* > 0.05) (Figure 5B).

ROC analysis indicated that plasma NF-L concentrations on D2 did not separate CI+ from CI− animals (AUC 0.579, *p* > 0.05) (Figure 5C).

### 2.7. Plasma NF-L as a Prognostic Biomarker for Post-Traumatic Epileptogenesis

Finally, we assessed whether, within the TBI group, plasma NF-L levels differentiated the epileptic (TBI+) from non-epileptic (TBI−) animals.

**Figure 6:**
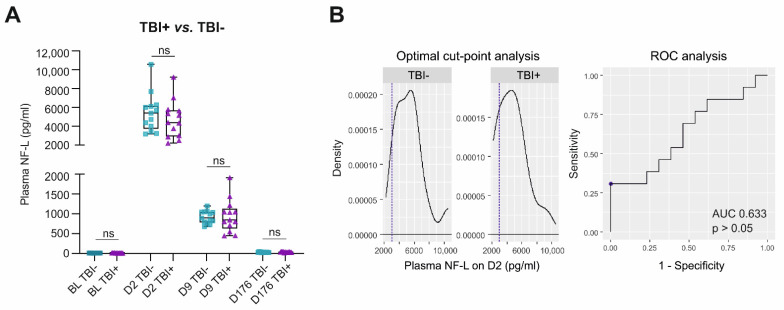
Plasma NF-L and epileptogenesis after TBI. (**A**) Box and whisker plots (whiskers: minimum and maximum; box: interquartile range; line: median) showing plasma NF-L levels (*y*-axis) in different groups and time-points (*x*-axis). Plasma NF-L levels on D2, D9, or D176 did not differ between rats that did (TBI+, n = 13) or did not develop epilepsy (TBI−, n = 13, *p* > 0.05). (**B**) Plasma NF-L levels on D2, D9, or D176 did not distinguish TBI+ and TBI− rats in the ROC analysis (*p* > 0.05, results for D2 shown in the figure). Statistical significance: ns, not significant (Mann-Whitney U test). Abbreviations: AUC, area under the curve; BL, baseline; D2, day 2 post-TBI; D9, day 9; D176, day 176; NF-L, neurofilament light chain; ROC, receiver operating characteristics.

***Plasma NF-L levels.*** Of the 26 rats with TBI, 13 had epilepsy (TBI+), and 13 did not (TBI−). Plasma NF-L levels did not differ between the TBI+ and TBI− groups at any time-point (Figure 6A). Also, the D2 to D9 change in the plasma NF-L concentrations did not differ between the TBI+ (mean decrease 3761 ± 1966 pg/mL, range 1434–8164 pg/mL) and TBI− (mean decrease 4523 ± 1966 pg/mL, range 2457–9527 pg/mL, *p* > 0.05) groups. Similarly, the D9–D176 change did not differ between TBI+ and TBI− (TBI+ mean decrease 911 ± 407 pg/mL, range 408–1863 pg/mL vs. TBI− mean decrease 885 ± 162 pg/mL, range 646–1166 pg/mL, *p* > 0.05). Within the TBI+ group, 3 of the 13 rats had a seizure on the day preceding the blood sampling; their NF-L levels, however, did not differ from those in other TBI+ rats (*p* > 0.05).

***Severity of epilepsy (clusters vs. no clusters).*** The total number of seizures did not correlate with plasma NF-L levels at any time point (*p* > 0.05). Of the 13 TBI+ rats, 4 had seizure clusters (≥3 seizures within 24 h). The plasma NF-L levels did not differ significantly between TBI+ rats with or without seizure clusters at any time-point (*p* > 0.05).

***ROC analysis.*** The plasma NF-L concentration did not distinguish TBI+ from TBI− animals on D2 (AUC 0.633, *p* > 0.05), D9 (AUC 0.521, *p* > 0.05), or D176 (AUC 0.479, *p* > 0.05) (ROC for D2 shown in Figure 6B).

## 3. Discussion

The present study investigated whether rat plasma NF-L levels predict structural and behavioral outcomes and post-traumatic epileptogenesis after lateral FPI-induced TBI. Our findings revealed that the higher the acute plasma NF-L levels, the larger the cortical lesion in acute MRI and chronically in histology. Also, the higher the acute plasma NF-L levels, the poorer the performance in the neuroscore test. In contrast, we found no association between plasma NF-L levels and somatomotor recovery, the development of chronic cognitive impairment, or epileptogenesis.

### 3.1. Presence and Temporal Profile of NF-L in the Plasma after TBI

Animal models of TBI enable detailed longitudinal studies on the temporal expression of potential plasma molecular biomarkers and how their levels relate to disease severity and progression [10]. Several studies have assessed circulating NF-L levels in rodent TBI models, including those induced by lateral FPI, single or repeated mild awake closed-head injury, and Marmarou’s weight-drop injury in rats [23,24,25,26], and a closed-head impact model of engineered rotational acceleration (CHIMERA) in mice [27]. All but one of these reports, however, focused on mild or mild-to-moderate TBI [25]. To the best of our knowledge, the present study is the first to assess circulating NF-L concentrations over time and their value as a preclinical prognostic biomarker for chronic functional outcomes after severe TBI induced by lateral FPI.

Plasma NF-L concentrations peaked on D2 after lateral FPI-induced TBI, reaching approximately 5000 pg/mL, or a 483-fold increase compared with the pre-injury levels. Although comparisons of injury severities and NF-L analyses between laboratories are challenging, plasma NF-L levels in our rats on D2 were comparable to those reported by Wong et al. [25]. Wong and co-workers also used SIMOA technology, assessed serum NF-L levels on D2 post-injury, and induced TBI in Sprague-Dawley rats with lateral FPI using a 2.6–3.0 atm impact force, comparable to the present study. Similar to our observations in the lateral FPI model, plasma NF-L concentrations in an awake closed-head injury model of mild TBI or Marmarou’s weight-drop model peaked on D1-2 post-injury [23,26]. As expected, however, the levels after severe TBI (5000 pg/mL) were substantially higher than those after a single mild TBI (~6 pg/mL) or mild-to-moderate weight drop (~60 pg/mL).

After a massive 483-fold increase on D2, the plasma NF-L levels declined rapidly to almost 89-fold on D9 compared with the baseline. Interestingly, even though the increase in the NF-L concentrations was substantially lower after mild than severe TBI, the temporal profile of the serum NF-L levels was comparable [23]. Similar to that after severe TBI, concentrations decreased from approximately 50 pg/mL on D1–2 to approximately 40 pg/mL on D7, and to 30 pg/mL on D14 after a single mild TBI [23].

Interestingly, even at 6 months post-injury, we detected an approximately 3-fold increase in NF-L levels. The average concentration was 32 pg/mL, which is close to the level observed after single or repeated mild TBI in rats [23,24]. Previous studies reported an approximately 3-week half-life of the NF-L protein in mouse brain tissue [28]. However, information is needed on the biological half-life of circulating NF-L in normal and/or injured rats.

Recent human data suggests that NF-L fragments are secreted by the kidney, and the filtration rate can affect the blood NF-L levels [29,30]. Although the clearance time of NF-L protein released into the blood in the rat lateral FPI model remains to be determined, it is likely to be less than 6 months, and therefore, the chronically increased levels suggest ongoing chronic release of NF-L from the brain tissue into the blood [19,31,32]. Our observations support previous histologic and diffusion tensor imaging studies reporting chronic ongoing axonal injury after severe lateral FPI-induced TBI [33,34]. Recently, a prolonged increase in post-injury serum NF-L levels was reported in 50% of rats exposed to a blast overpressure model at 4 months post-injury, supporting the idea that a chronic increase in post-injury plasma NF-L levels is not model-specific [35]. Importantly, studies in human TBI have also reported increased serum NF-L levels up to 5 years after injury [19].

Studies of patients with different types of TBI indicate an injury severity-dependent increase in serum levels of NF-L up to 100–200 pg/mL when sampled within 24 to 48 h post-admission [16,36]. Interestingly, the D2 serum NF-L levels were substantially lower in humans than in rats after severe TBI, approximately 200 pg/mL vs. 5000 pg/mL in rats [16]. In rats, we observed a large reduction in NF-L levels from the D2 value of 5000 pg/mL down to 900 pg/mL on D9. Instead, in humans, the serum NF-L levels continue to increase during the 2nd post-injury week, reaching a level of approximately 2000 pg/mL on days 10–12 after admission [15,16]. Shahim and co-workers recently reported that at 30 days after TBI, the median serum NF-L levels were approximately 13 pg/mL—almost 2-fold higher than those of controls [19]. These studies suggest a different temporal evolution in the post-injury levels of circulating NF-L in rats and humans.

Taken together, while the magnitude of the plasma/serum NF-L concentrations after TBI is greater and the timescale of expression is shorter in rat models than in humans, the response to injury severity appears similar in rats and humans with TBI. In both rats and humans, however, the levels remain higher for a prolonged period of time, suggesting ongoing axonal injury.

### 3.2. Acute Plasma NF-L Levels Report on Cortical Lesion Severity in Structural MRI and Histology

Despite the heterogeneity of the clinical population, timing of blood sampling, and MRI analysis, recent clinical studies demonstrated an association between increased serum NF-L levels and the severity of brain damage as well as the progression of brain pathology [17,19]. Our correlation analysis revealed that the higher the plasma NF-L levels on D2, the greater the cortical lesion volume when imaged within hours after the blood sampling on the same day. The D2 NF-L levels, however, did not predict lesion volume on D7 or D21. This was expected, as we previously reported that the volume of the abnormal cortical T_2_ area undergoes dynamic changes during the first post-injury weeks in rats with lateral FPI, becoming significantly reduced from D2 to D7 and increasing thereafter [37]. Importantly, a correlation was detected between the acute D2 NF-L levels and the cortical lesion area at 6 months post-TBI. To the best of our knowledge, this is the first study assessing the relation between circulating NF-L levels and cortical lesion severity and progression in rats with severe TBI.

The results suggest that, similar to humans, plasma NF-L levels report on the severity of acute TBI and its chronic progression in the lateral FPI model.

### 3.3. Elevated Plasma NF-L as a Diagnostic Biomarker for TBI and Craniotomy in the Rat Lateral FPI Model

Circulating NF-L is considered a diagnostic biomarker for mild and severe TBI in clinical studies [16,19,38]. Next, we analyzed the sensitivity and specificity of post-TBI plasma NF-L levels as diagnostic biomarkers for TBI after lateral FPI-induced severe TBI. We found that plasma NF-L differentiated TBI rats from naïve controls both at the acute (D2 and D9) and chronic (6-month) time points. Our study expands the previous results by Wong et al. [25] who reported comparable results at the D2 post-TBI time-point. Increased circulating NF-L levels were also reported after a single or repeated mild TBI in animal models, but the sensitivity and specificity of plasma/serum NF-L as a diagnostic biomarker for mild TBI remain to be assessed [23,24].

Unexpectedly, we observed that the plasma NF-L levels increased from 9.6 pg/mL at baseline to 244 pg/mL on D2 after craniotomy in sham-operated animals. Moreover, plasma NF-L levels separated sham controls from naïve rats on D2 with an AUC of 1.0 and a cut-off concentration of 49 pg/mL. Previous studies demonstrated that craniotomy can induce an inflammatory reaction in the underlying cortex [39]. Our MRI analysis suggested mild T_2_ relaxation abnormalities in sham-operated controls with a craniotomy. Interestingly, however, the greatest T_2_ area increase was located rostral to the craniotomy center and close to the rhinal fissure rather than under the craniotomy. We also found a parasagittal signal decrease on D7 in a region corresponding to the medial aspect of the craniotomy. Plasma NF-L levels, however, were not associated with the severity of the T_2_ abnormality. We assume that the increased plasma NF-L levels are related to craniotomy-induced meningeal irritation and submeningeal inflammation and subtle intracortical axonal injury rather than to the cortical cellular pathology suggested by T_2_ MRI.

Taken together, not only TBI of different severities, but also a mere craniotomy, typically used as a sham procedure, can lead to an increase in the plasma NF-L levels. This observation emphasizes the importance of using naïve animals as “controls” in biomarker discovery studies, especially when one examines low-expressing biomarkers or milder injury severities with lower biomarker expression.

### 3.4. Increased Plasma NF-L Levels Correlate with Acute Somatomotor Impairment but Not with Recovery after TBI

Previous studies demonstrated that the greater the severity of the brain injury, the greater the somatomotor impairment [40]. Here, we used the composite neuroscore test to assess the severity of the somatomotor impairment and recovery during the first 2 weeks after lateral FPI. We found that the higher the plasma NF-L concentration on D2, the greater the impairment in the neuroscore test on D2. The D2 plasma NF-L levels, however, did not predict the performance on later follow-up points (D6 and D14). Consistent with our findings, previous studies of rats with single or repeated mild TBI indicated that the higher the acute serum NF-L levels, the greater the impairment in the beam-walking test [23,24].

Like the severity of impairment on the D2 neuroscore test, the trajectory of recovery also varied between animals. We were unable to find any association between the D2 plasma NF-L levels and early recovery, however, which was measured as a change in the neuroscore from D2 to D6. The NF-L levels also did not differ between the late-recovering and non-recovering animals.

Taken together, increases in the plasma/serum NF-L levels indicate not only the severity of brain damage, but also the severity of the acute somatomotor impairment. The correlation between the D2 somatomotor impairment and elevation in plasma NF-L levels may relate to axonal injury in pathways required for proper somatomotor performance.

### 3.5. Increased Plasma NF-L Levels Do Not Differentiate Animals with or without Chronic Hippocampus-Dependent Memory Impairment after TBI

In chronic neurodegenerative disease and multiple sclerosis, high NF-L levels are associated with cognitive impairment [41,42,43,44]. Rats with severe lateral FPI-induced TBI show hippocampus-dependent memory deficits already on D2 after TBI and remain impaired for months [45]. Moreover, animals show both hippocampal principal cell and interneuron death, with remarkable atrophy in hippocampal afferent and efferent myelinated axonal pathways after lateral FPI that progresses over weeks to months post-injury [46,47,48]. Therefore, we anticipated that acutely increased plasma NF-L levels would differentiate rats that will develop chronic memory impairment from those who will not. Our cut-point analysis of the entire EPITARGET animal cohort indicted that 70% of rats with severe TBI had poor memory on D37. In the sub-cohort, the percentage was 73%, indicating no animal selection bias. In contrast to our expectations, we detected no difference in the plasma NF-L levels between rats with or without cognitive impairment.

Further studies are needed to explore the cerebral origin of elevated post-TBI NF-L plasma levels to determine whether plasma NF-L has any localizing specificity, such as to lesions in selective myelinated axonal pathways, rather than being a nonspecific marker of axonal injury.

### 3.6. Elevated Plasma NF-L Levels Do Not Predict Post-Traumatic Epileptogenesis

Previous studies reported that the risk of PTE relates to injury severity [49] and that the epileptogenic focus develops at the perilesional cortex [50]. As circulating NF-L levels reflect the extent of cortical injury, we investigated whether an acute NF-L increase predicted which animals developed PTE during the 6-month follow-up. We failed to detect any differences in plasma NF-L levels at any investigated time point between the rats that developed PTE and those that did not.

Previous studies demonstrated that the average interictal NF-L levels in patients with drug-refractory epilepsy due to structural and other etiologies, genetic epilepsy, or temporal lobe epilepsy with hippocampal sclerosis are comparable to those in controls, although they can be slightly increased in some subpopulations of patients when using a cut-off level of 10 pg/mL [51,52,53]. Other studies, however, reported that patients with post-stroke epilepsy or epilepsy related to auto-immune encephalitis have chronically increased serum NF-L levels, even though the levels remain below 100 pg/mL [52,54].

In addition to etiology, prior seizure occurrence could also influence the NF-L levels. Nass et al. [55] reported a non-significant increase (<1 pg/mL) in serum NF-L levels caused by a single tonic-clonic seizure when assessed right after the seizure that remained quite stable over the next 24 h. Also, no change in NF-L levels was detected in children with simple or complex febrile seizures or epileptic seizures when serum was analyzed within 2 h after seizure onset [56]. In our NF-L animal cohort, 50% of the rats in the TBI group experienced 1-17 seizures over the 1-month video-EEG monitoring period in the 6th post-injury month and were diagnosed with PTE. Consistent with clinical studies, the NF-L levels at 6 months post-TBI did not differ between rats with or without epilepsy. Also, there was no correlation between the NF-L levels and seizure frequency. Nor did we find a difference in NF-L levels between epileptic rats with or without seizure clusters (i.e., ≥3 seizures/24 h). Of the 13 rats with epilepsy, 3 had experienced an unprovoked seizure on the day preceding the plasma sampling. Their NF-L concentrations, however, did not differ from those of the other animals. These findings suggest that the epilepsy- and seizure-related increases in plasma NF-L are meager in the lateral FPI model compared with the TBI-induced increase in circulating NF-L levels, thereby reproducing the data available on structural epilepsies in humans to date.

A recent study reported Increased serum NF-L levels in patients with status epilepticus (SE) when assessed within 48 h after the beginning of seizure activity [51]. In addition, elevated NF-L levels are associated with the development of treatment refractoriness and 30-day clinical worsening or death. We previously reported that 90% of rats with lateral FPI develop SE after TBI, lasting 3 to 4 days [57]. Thus, the ongoing epileptiform activity could have affected the D2 plasma NF-L levels in our study cohort. Unfortunately, we did not perform acute vEEG recordings in these animals. We consider it unlikely, however, that the occurrence of SE was a major contributor to the NF-L levels on D2. In humans, the SE-induced increase measured within 48 h after the start of SE, corresponding to the timeline of D2 plasma sampling in the present study, ranged from 13 pg/mL to 101 pg/mL [51]. In our animal cohort, the NF-L levels increased from 11 pg/mL to 5072 pg/mL, supporting the view that TBI rather than SE was the major contributor to the robust NF-L increase. Considering the slower kinetics of the brain injury-induced increase in serum NF-L in humans compared with that in rats, further clinical studies with more chronic sampling time-points are needed for more accurate comparisons between the clinical and experimental studies.

Taken together, acute post-TBI plasma NF-L levels did not predict the development of PTE or correlate with the severity of PTE after lateral FPI.

### 3.7. Methodologic Considerations

Human studies suggest a mild, 3% average age-related increase in circulating NF-L levels, which is proposed to relate to subclinical co-morbidities [58]. In our study cohort, the first sampling, which included naïve and control samples, was performed in animals at the age of 3 months, whereas the last sampling point (D176) was at the age of 9 months. Although we cannot completely rule out that some of the 20 pg/mL concentration difference between the 3 and 9 months samples in the TBI group relate to aging rather than continuing axonal damage, we consider an aging effect unlikely as it had required an almost 20% monthly age-related increase in NF-L levels. Also, as all samples were assessed in the same batch, the assay-related variability does not explain the finding.

The average peak post-TBI concentration of plasma NF-L in rats was approximately 5000 pg/mL, whereas in humans with severe TBI, the median concentrations were approximately 2000 pg/mL, with some subjects showing levels up to 10,000 pg/mL or even higher [15,16]. It remains to be explored whether the ~2.5-fold higher average circulating NF-L concentrations reflect a greater proportion of brain damage in rodents than in humans, differences in the volume distribution or NF-L metabolism, or other factors. Finally, the kinetics of released NF-L flow to cerebrospinal fluid and blood compartments and its relation to the breakdown of blood-CSF and blood brain barriers in a given subject need to be further explored [59].

Finally, the study was powered to differentiate the TBI+ and TBI− groups, if the AUC of the biomarker was 0.800 or greater. However, the data presented for the smaller subgroups should be interpreted as preliminary observations due to the small sample size.

## 4. Materials and Methods

### 4.1. Animals

**Figure 7:**
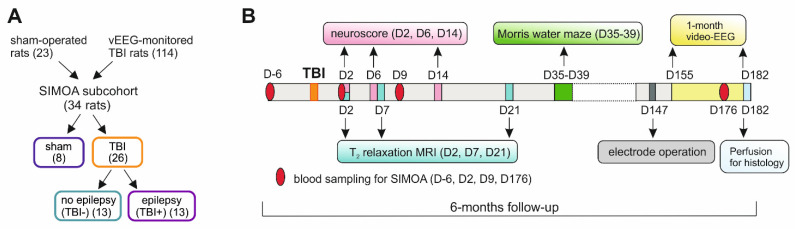
Study design. (**A**) The first 34 rats [8 sham-operated experimental controls, and 26 with traumatic brain injury (TBI)] completing the 6-month follow-up in the EPITARGET animal cohort of 137 animals (23 sham, and 114 TBI) were included in the present analysis [22,37]. Within the TBI group, 13 rats exhibited no unprovoked seizures in video-encephalogram (vEEG) (TBI−). In 13 rats, we found at least 1 unprovoked seizure during the 6th month vEEG (TBI+). The number of animals was based on a power calculation. That is, we expected the plasma NF-L to separate the TBI− and TBI+ groups with AUC 0.800 (MedCalc software). (**B**) Timing of the tests included in the present analysis (for a complete study design, see [22]). During the 6-month follow-up, rats underwent blood sampling via the tail vein, behavioral tests (neuroscore and Morris water maze), in vivo quantitative T_2_ magnetic resonance imaging (MRI), and 1-month continuous vEEG monitoring. Plasma levels of neurofilament light chain were assessed using a single molecule array (SIMOA). Abbreviations: D, day.

The study design is summarized in Figure 7. The study cohort included the first 34 rats (8 sham, 26 TBI) of a total of 137 animals (23 sham-operated experimental controls, 114 TBI) that completed the 6-month-long EPITARGET project (https://epitarget.eu, accessed on 4 October 2022). Detailed descriptions of the project and procedures were previously reported [22,37].

The cohort size for the NF-L analysis was calculated based on an expected area under the curve (AUC) of 0.800 (power 0.8, *p* < 0.05, epilepsy vs. no-epilepsy ratio of 1:1, MedCalc software). Consequently, plasma NF-L was analyzed from 34 adult male Sprague-Dawley rats (Envigo Laboratories S.r.l, Udine, Italy), 8 of which were sham-operated controls (to assess the direction and magnitude of the changes after TBI), and the remaining 26 were exposed to lateral fluid-percussion (FPI)-induced TBI [including 13 rats that developed epilepsy (TBI+) and 13 that did not develop epilepsy (TBI−)].

At the time of injury or sham operation, average body weight was 364 g ± 13 g (median 364 g, range 337–396 g). Rats were housed in individual cages in a controlled environment (temperature 22 ± 1 °C, humidity 50–60%, lights on 07:00–19:00), and had free access to food and water. All experiments were approved by the Animal Ethics Committee of the Provincial Government of Southern Finland and performed in accordance with the guidelines of the European Community Council Directives 2010/63/EU.

### 4.2. Lateral Fluid-Percussion-Induced Traumatic Brain Injury

TBI was induced by lateral FPI (as described in detail in [22]). Impact pressure in the EPITARGET cohort was adjusted to produce severe TBI with an expected post-impact mortality of 20–30% within the first 48 h. The mean impact pressure in the NF-L cohort was 3.27 ± 0.07 atm (*n* = 26, median 3.26 atm, range 3.13–3.41 atm). Time spent in apnea and the occurrence and duration of impact-related seizure-like behaviors were monitored and documented. The sham-operated experimental controls underwent the same anesthesia and surgical procedures without the induction of lateral FPI.

### 4.3. Analysis of Plasma NF-L Levels

***Plasma sampling.*** NF-L was analyzed in plasma samples collected at baseline (7 days before injury [range D-8 to D-5, mean D-6]), D2 (48 h), D9, and at 6 months (D174 to D178, mean D176) after injury or sham operation. Sampling of tail vein blood and preparation of plasma samples were performed according to van Vliet et al. [60]. Briefly, rats were placed in an anesthesia chamber and anesthetized with 5% isoflurane. Anesthesia was maintained with 1–2% isoflurane through a nose mask. Blood was drawn from the lateral tail vein into 2 Microtainer K_2_ EDTA-tubes (#365975, di-potassium ethylenediaminetetraacetic acid, BD Microtainer, BD Biosciences, Franklin Lakes, NJ, USA), 500 μL of blood per tube, using a 24G butterfly needle. Within 1 h after blood sampling, blood tubes were centrifuged at 1300× *g* for 10 min at 4 °C (5417R Eppendorf Biotools). Plasma aliquots of 50 µL were carefully collected, pipetted into 0.5-mL Protein LoBind tubes (#022431064, Eppendorf AG, Hamburg, Germany), and stored at −70 °C.

***Single molecule array of NF-L.*** Plasma NF-L levels from 26 TBI rats [13 that developed epilepsy (TBI+) and 13 that did not develop epilepsy (TBI−)] were analyzed at baseline, and on D2, D9, and D176. In sham-operated experimental controls, we analyzed the plasma collected at baseline and on D2. The baseline plasma of both sham-operated and TBI rats was considered to represent the plasma of naïve animals.

Plasma NF-L levels were measured using a Single Molecule Array (SIMOA) digital immunoassay (NF-light Advantage assay, #103186, Quanterix, Lexington, MA, USA) [61]. Prior to SIMOA, the plasma samples were diluted 1:16 in NF-light sample diluent buffer (Quanterix). All samples were analyzed using the same batch of reagents and the same SIMOA HD-1 instrument (Quanterix). The average intra-assay coefficient of variability for duplicate sample measurements was 4.5%. Three D2 samples had an average enzyme per bead (AEB) value beyond the range of the calibration curve (>7200 pg/mL), and their concentrations were determined by extrapolation [62]. To assess the possible effect of hemolysis on NF-L levels, absorbance at 414 nm was measured in each sample using a NanoDrop spectrophotometer (NanoDrop 1000, Thermo Fisher Scientific, Waltham, MA, USA).

### 4.4. Behavioral Tests

#### 4.4.1. Composite Neuromotor Score (Neuroscore)

The composite neuroscore test was used to measure the severity of somatomotor and vestibular deficits [40]. The tests were performed at D-6, D2, D6, and D14 (for details, see [22]). Briefly, the test included 7 parameters: (1) left and right forelimb flexion (2 parameters), (2) left and right hindlimb flexion (2 parameters), (3) left and right lateral pulsion resistance test (2 parameters), and (4) angle board standing test (1 parameter). The animals were scored from 0 (severely impaired) to 4 (normal) on an ordinal scale for each parameter, resulting in a composite neuroscore of 0 to 28 (the maximum score).

To evaluate the rate of early (D2 to D6), late (D6 to D14), and overall (D2 to D14) somatomotor recovery, the recovery index was calculated for each TBI rat as a percentage difference in the neuroscore between time-points. Rats with a D6/D2 recovery index > 150% were classified into the “good early recovery” group, and those with an index ≤ 150% were classified into the “poor early recovery” group. Rats with a D14/D6 recovery index > 100% were classified into the “late recovery” group, and those with an index of ≤100% were classified into the “poor late recovery” group. Rats with a D14/D2 recovery index > 200% were classified into the “good overall recovery” group, and those with an index ≤ 200% were classified into the “poor overall recovery” group.

#### 4.4.2. Morris Water Maze

The Morris water-maze test was used to assess spatial learning and memory. The tests, including a probe trial, were performed on D35–D39 after injury (for details, see [22]).

Cognitive performance of rats with TBI varied substantially on D35–D39, with some exhibiting severe impairment and others performing at control level [22]. Therefore, we next categorized the TBI rats (n = 118) into those that showed or did not show memory impairment compared with the sham-operated controls (n = 23) by performing a cut-point analysis. To maximize the statistical power, the data used in the cut-point analysis was derived from the entire EPITARGET cohort. Based on the cut-off value of the latency to find the hidden platform on the 3rd testing day, we categorized each rat into either the “cognitively impaired” (CI+) or the “cognitively not impaired” (CI−) group.

### 4.5. Magnetic Resonance Imaging (MRI) and Lesion Analysis

Details of the quantitative T_2_ MRI performed in the EPITARGET cohort, including the rats analyzed here, were previously provided [37]. Rats were imaged on D2, D7, and D21 after injury (Figure 7B).

***Cortical lesion volume.*** Analysis of cortical lesion volumes from MRI was previously described [37]. Briefly, T_2_ relaxation time maps were estimated from multi-slice-multi-echo spin-echo images acquired with a 7-Tesla Bruker PharmaScan magnet (Bruker BioSpin MRI GmbH). The imaging time-points were 2, 7, and 21 days after TBI. The slice thickness was 500 µm, in-plane resolution 201 × 201 µm^2^, repetition time 3016 ms, and echo times 14.6, 29.2, 43.8, 58.4, 73.0, and 87.6 ms. As a result of the challenges in the image registration accuracy caused by cortical lesions, the injured cortex was manually outlined for each animal at each time-point. Based on their T_2_ values, imaging voxels within the cortex were classified as normal or abnormal. The range for normal T_2_ values was defined as 45 ms ≤ T_2_ ≤ 55 ms, with the lower limit corresponding to the 2.5th percentile and the upper limit corresponding to the 97.5th percentile of all imaging voxels of all sham-operated controls across all time-points. Values below the lower limit or above the upper limit were classified as abnormal. The number of abnormal voxels was counted and multiplied by voxel size to obtain an estimate of the cortical lesion volume for each animal.

**Figure 8:**
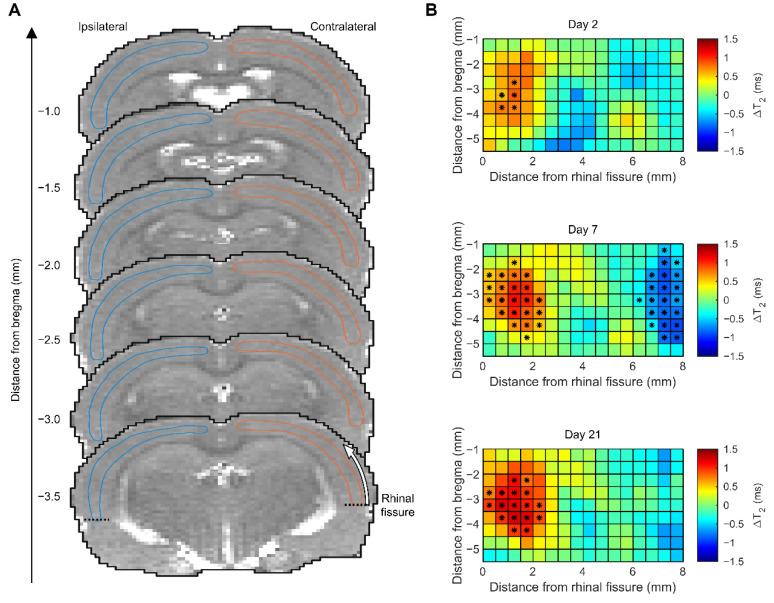
Cortical T_2_ magnetic resonance imaging (MRI) analysis in the sham-operated experimental control group. (**A**) T_2_ images of the sham animals were registered to a template brain. Then, the ipsilateral (left, blue outline) and the contralateral (right, orange outline) cortex was manually outlined in each image slice (thickness 0.5 mm). In each slice, a cortical profile of the T_2_ relaxation time was measured, starting at the rhinal fissure and continuing dorsally towards the brain midline as indicated by the white arrow (bottom slice). (**B**) The cortical T_2_ profiles of different slices were combined, filtered using a 2-dimensional isotropic Gaussian filter with a standard deviation of 0.5 mm, and interpolated on a grid with a 0.5 × 0.5 mm^2^ resolution. We found an ipsilateral signal increase on D2, D7, and D21 in areas close to the rhinal fissure at the rostrocaudal level (-3.5 mm from the bregma) as the median of the ipsilateral T_2_ area was increased compared with that contralaterally. We also found a parasagittal signal decrease on D7. Color coding of the heatmap: red indicates a positive ipsilateral vs. contralateral difference in T_2_ (higher T_2_ ipsilaterally), and blue indicates a negative difference (higher T_2_ contralaterally). An asterisk (*) at a given grid point indicates a statistically significant difference for the median T_2_ (Mann-Whitney U-test, after correcting for multiple comparisons using the Benjamini-Hochberg procedure with an average false discovery rate of 0.05).

***Gridded unfolded cortical map*.** As we unexpectedly found increased NF-L levels in the D2 plasma of sham-operated animals (see results), we analyzed their D2 T_2_ MRI in further detail to identify the locations of possible structural abnormalities. No major cortical lesions were detected, and we were able to accurately register the images to a template brain (see [63]). First, the ipsilateral and contralateral cortex were manually outlined in each 0.5-mm-thick imaging slice in the template brain. Then, unfolded cortical maps were generated from the registered images as described previously [37]. The cortical profile of T_2_ was measured in each slice, starting at the rhinal fissure and continuing toward the brain midline (Figure 8A). The T_2_ profiles of different slices were joined to form a 2-dimensional mapping of T_2_ relaxation times over the cortical surface. The resulting 2-dimensional cortical maps were filtered using an isotropic spatial Gaussian filter (standard deviation 0.5 mm), followed by an interpolation of the maps on a grid with a 0.5 × 0.5 mm^2^ resolution. The Mann-Whitney U-test along with the Benjamini-Hochberg procedure (average false discovery rate of 0.05) [64] were used to correct for multiple comparisons and to test for differences between the ipsilateral and contralateral cortical T_2_ values at each grid point.

### 4.6. Video-EEG Monitoring

At 5 months post-TBI (D147), rats were anesthetized and implanted with 3 skull electrodes. Starting on D154 (1 week after electrode implantation), rats underwent continuous (24/7) video-EEG (vEEG) monitoring for 4 weeks to diagnose PTE [22]. Rats were defined as having epilepsy if at least one unprovoked electrographic seizure was detected (Supplementary Figure S4). The electroencephalographic seizure was defined as a high-amplitude rhythmic discharge that clearly represented an atypical EEG pattern (i.e., repetitive spikes, spike-and-wave discharges, poly-spike-and-wave discharges, or slow-waves with frequency and amplitude modulation) that lasted >10 s [65]. The prevalence of epilepsy in the EPITARGET cohort was 27% (31/114).

### 4.7. Histology and Preparation of Cortical Unfolded Maps

***Perfusion.*** To assess the location and extent of the FPI and exclude non-TBI related epileptogenic lesions (e.g., abscess), rats were intracardially perfused for histology after completing the vEEG (D182). Briefly, rats were deeply anesthetized with pentobarbital (60 mg/kg, i.p.) and perfused transcardially with 0.9% NaCl followed by 4% paraformaldehyde in 0.1 M sodium phosphate buffer (PB), pH 7.4. The brain was removed from the skull, fixed in 4% paraformaldehyde for 4 h, cryoprotected in 20% glycerol in 0.02 M potassium phosphate-buffered saline (KPBS, pH 7.4) for 24 h, frozen in dry ice, and stored at −70 °C for further processing.

Frozen coronal sections of the brain were cut (25-µm thick, 1-in-12 series) using a sliding microtome. The first series of sections was stored in 10% formalin at room temperature and used for thionin staining. Other series of sections were collected into a tissue collection solution (30% ethylene glycol and 25% glycerol in 0.05 M PB) and stored at −20 °C until processed.

***Nissl staining.*** The first series of sections was stained with thionin, cleared in xylene, and cover-slipped using Depex^®^ (BDH Chemical, Poole, UK) as a mounting medium.

***Preparation of cortical unfolded maps.*** To assess the cortical lesion area and the damage to different cytoarchitectonic cortical areas after TBI, thionin-stained sections were digitized (40×, Hamamatsu Photonics, NanoZoomer-XR, NDP.scan 3.2). Unfolded cortical maps were then prepared from the digitized histologic sections as described in detail by [66] and by applying in-house software from https://unfoldedmap.org (accessed on 4 October 2022) adapted to the rat brain [67].

### 4.8. Statistical Analysis

Data were analyzed using GraphPad Prism 9 and RStudio (v. 1.1.463) by R (v. 4.0.2). Differences in the mean NF-L plasma levels between groups were assessed with the Mann-Whitney U test (unpaired data) or the Wilcoxon matched-pairs signed rank test (paired data). Differences in mean NF-L plasma levels in TBI rats over different time points were assessed by the Friedman test followed by the Wilcoxon *post hoc* test. Correlations between the plasma NF-L levels and behavioral outcome measures or lesion size in MRI or histology were assessed with the Spearman rank correlation test (ρ). Evolution of the composite neuroscore over the testing period was assessed by the Friedman test followed by the Wilcoxon *post hoc* test. Categorization of rats with TBI into those with or without cognitive impairment (CI+ or CI−, respectively) on D35-D39 was performed with the cut-point analysis, using the cutpointr package in R. The optimal cut-off value for each parameter was defined as the maximal sum of sensitivity and specificity. The sensitivity and specificity of plasma NF-L as a prognostic biomarker for cognitive impairment and epileptogenesis were assessed with receiver operating characteristic (ROC) analysis using the pROC package in R. Statistical significance of the AUC was evaluated by the Mann-Whitney U test. A *p*-value less than 0.05 was considered significant. Data are expressed as the mean ± standard deviation of the mean (SD).

## 5. Conclusions

This is the first systematic study of plasma NF-L levels in a model of PTE with structural, behavioral, and vEEG follow-up. Our data show that TBI and even a craniotomy lead to an increase in plasma NF-L levels. After lateral FPI, the higher the NF-L levels, the greater the cortical brain damage and somatomotor impairment. We did not find an association between plasma NF-L levels and the evolution of chronic hippocampus-dependent memory impairment. Nor did we find a correlation between plasma NF-L and somatomotor recovery or epileptogenesis. Our data indicate that although the dynamics of post-injury plasma NF-L levels seem faster in the rat model compared with that in human TBI, the association of plasma NF-L with TBI-related factors such as severity of TBI and brain damage and magnitude of functional impairments is similar between the rat model and human TBI. Our data show that plasma NF-L is a sensitive biomarker to monitor post-injury structural and functional severity after lateral FPI and could serve as a promising noninvasive response biomarker in preclinical treatment trials.

## Data Availability

The data presented in this study are available on request from the corresponding author.

## Supplementary Materials

The following supporting information can be downloaded at: https://www.mdpi.com/article/10.3390/ijms232315208/s1.
