# Supplementary material for: Plasma Neurofilament Light Chain (NF-L) Is a Prognostic Biomarker for Cortical Damage Evolution but Not for Cognitive Impairment or Epileptogenesis Following Experimental TBI
Source: Int J Mol Sci. 2022 Dec 2;23(23):15208. doi: 10.3390/ijms232315208 (PMC9736117; doi:10.3390/ijms232315208)
Supplement: Supplementary file 1 [file ijms-23-15208-s001.zip › ijms-1981907-supplementary.pdf]

Supplementary Data

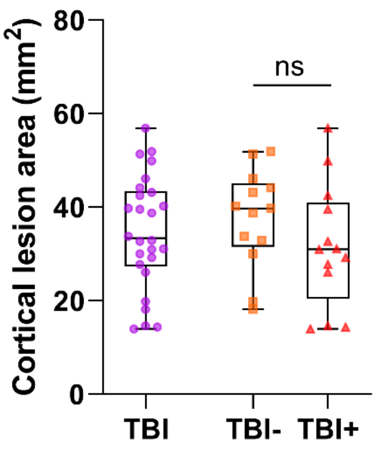

Supplementary Figure S1. **Lesion area in unfolded cortical maps at 6 months post-TBI.** Box and whisker plots (whiskers: minimum and maximum; box: interquartile range; line: median) showing the lesion area in unfolded cortical maps at 6 months (D182) after TBI of the rats included in the NF-L analysis. Average cortical lesion area in the TBI group (n=26) was 35.6 ± 12.2 mm^2. Within the TBI group, average cortical lesion area was 37.7 ± 10.6 mm^2 for rats without epilepsy (TBI-, n=13) and 31.5 ± 13.3 mm^2 for rats with epilepsy (TBI+, n=13). No difference was detected between the TBI+ and TBI- groups (Mann-Whitney U test, $p > 0.05$). Abbreviations: D, day; ns, not significant; TBI, traumatic brain injury.

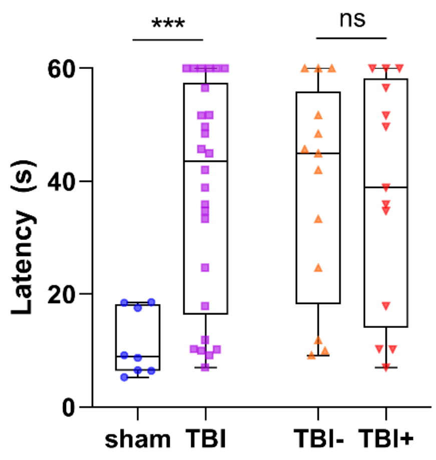

Supplementary Figure S2. Performance of NF-L cohort animals in Morris water-maze. Box and whisker plots (whiskers: minimum and maximum; box: interquartile range; line: median) showing the latency to find the hidden platform in the Morris water-maze test on D37 in 34 rats (8 sham, 26 TBI) included in the NF-L analysis. The latency was longer in rats with TBI than in sham-operated controls (38.3 s ± 19.4 s *vs.* 11.3 s ± 5.8 s, $p < 0.001$). Within the TBI group, no difference was found between the rats with (TBI+, n=13) or without epilepsy (TBI-, n=13) (37.9 s ± 25.5 s *vs.* 38.6 s ± 19.1 s, $p > 0.05$). Statistical significances: Mann Whitney U test, ***, p < 0.001; ns, not significant (p > 0.05). Abbreviations: TBI; traumatic brain injury.

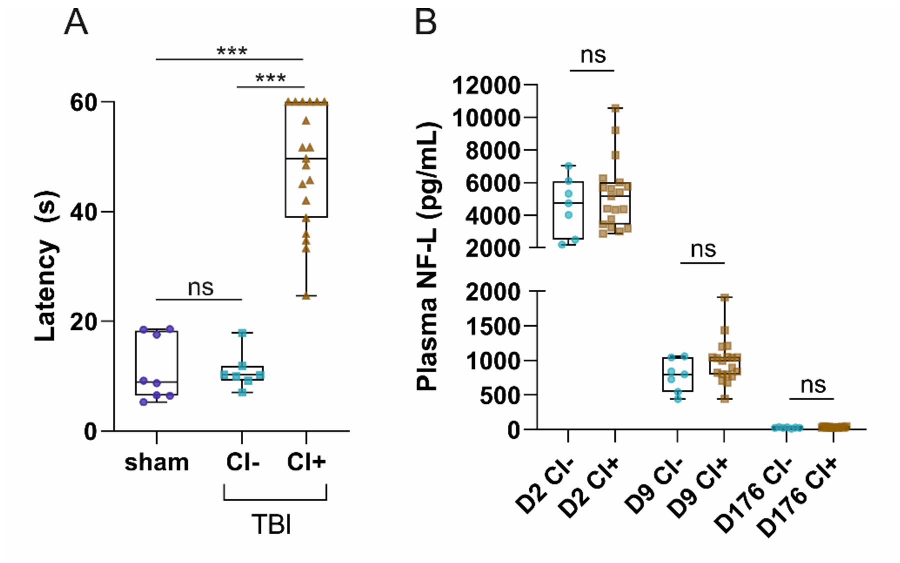

Supplementary Figure S3. Performance in Morris water-maze and plasma NF-L levels in cognitively impaired (CI+) and non-impaired (CI-) groups. (A) Box and whisker plots showing the latency to find the hidden platform in the Morris water-maze test on D37 in 34 rats (8 sham, 26 TBI) included in the NF-L analysis. The TBI rats were classified into cognitively non-impaired (CI-, n=7) and cognitively impaired (CI+, n=19) groups (cut-off latency value 19.2 s, see Fig. 7). Performance of CI- rats did not differ from that of sham-operated controls (*p* > 0.05). **(B)** Box and whisker plots showing that plasma NF-L levels were comparable in the CI-(n=7) and CI+ (n=19) groups at all three time points (*p* > 0.05). Statistical significances: Mann Whitney U test, ***, p < 0.001; ns, not significant (p > 0.05). Abbreviations: D2, day 2; D9, day 9; D176, day 176 after TBI; TBI, traumatic brain injury.

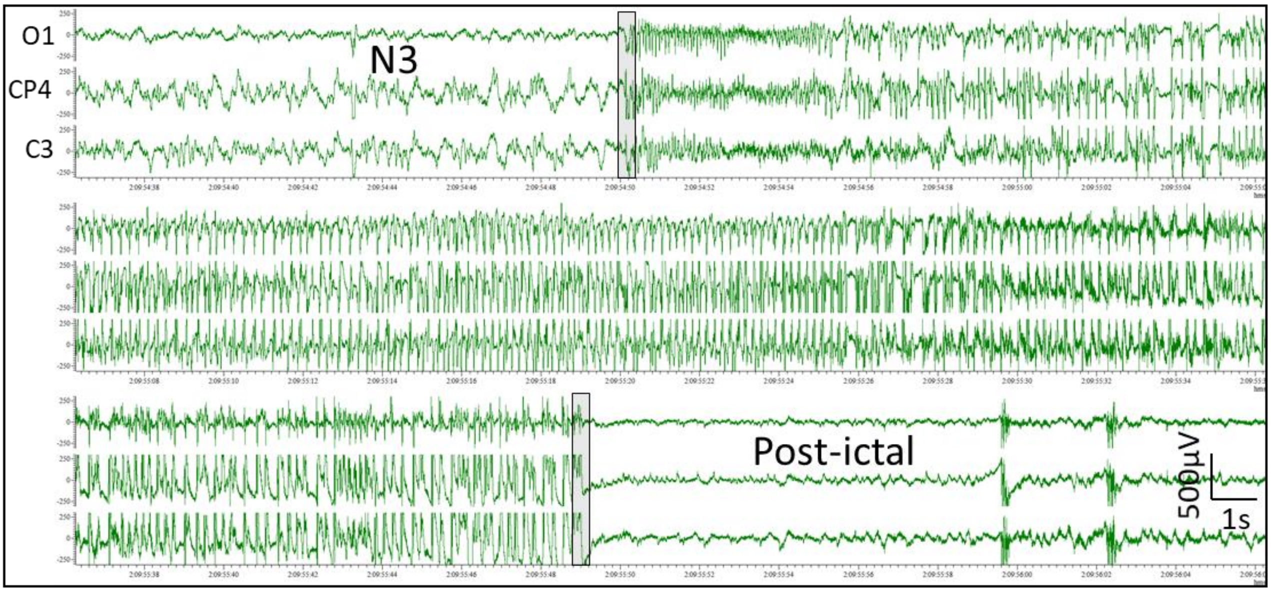

Supplementary Figure S4. A representative example of an electrographic seizure during the 4-weeks continuous video-EEG-monitoring on the 6th post-injury month. Electrode positions are labeled as C3 (ipsilateral frontal), CP4 (contralateral centroparietal) and O1 (ipsilateral occipital). Shaded boxes indicate the beginning and end of the seizure. Note that the seizure started at the N3 sleep and was followed by postictal attenuation in EEG. Abbreviations: N3, N3 sleep stage. Time scale is on x-axis and voltage (500 µV) on y-axis.
